# New signatures of the spin gap in quantum point contacts

**DOI:** 10.1038/s41467-020-19895-3

**Published:** 2021-01-04

**Authors:** K. L. Hudson, A. Srinivasan, O. Goulko, J. Adam, Q. Wang, L. A. Yeoh, O. Klochan, I. Farrer, D. A. Ritchie, A. Ludwig, A. D. Wieck, J. von Delft, A. R. Hamilton

**Affiliations:** 1 School of Physics, University of New South Wales, Sydney, NSW 2052 Australia; 2 ARC Centre of Excellence in Future Low-Energy Electronics Technologies, University of New South Wales, Sydney, NSW 2052 Australia; 3 Department of Physics, University of Massachusetts, Boston, MA 02125 USA; 4 Cavendish Laboratory, University of Cambridge, Madingley Road, Cambridge, UK; 5 Department of Electronic and Electrical Engineering, University of Sheffield, Sheffield, UK; 6 Angewandte Festkörperphysik, Ruhr-Universität Bochum, D-44780 Bochum, Germany; 7 Arnold Sommerfeld Center for Theoretical Physics, Ludwig-Maximilians Universität, München, Theresienstrasse 37, D-80333 München, Germany

**Keywords:** Nanowires, Spintronics, Quantum physics

## Abstract

One dimensional semiconductor systems with strong spin-orbit interaction are both of fundamental interest and have potential applications to topological quantum computing. Applying a magnetic field can open a spin gap, a pre-requisite for Majorana zero modes. The spin gap is predicted to manifest as a field dependent dip on the first 1D conductance plateau. However, disorder and interaction effects make identifying spin gap signatures challenging. Here we study experimentally and numerically the 1D channel in a series of low disorder p-type GaAs quantum point contacts, where spin-orbit and hole-hole interactions are strong. We demonstrate an alternative signature for probing spin gaps, which is insensitive to disorder, based on the linear and non-linear response to the orientation of the applied magnetic field, and extract a spin-orbit gap *ΔE* ≈ 500 *μ*eV. This approach could enable one-dimensional hole systems to be developed as a scalable and reproducible platform for topological quantum applications.

## Abstract (web summary)

In one-dimensional systems, the combination of a strong spin-orbit interaction and an applied magnetic field can give rise to a spin-gap, however experimental identification is difficult. Here, the authors present new signatures for the spin-gap, and verify these experimentally in hole QPCs.

## Introduction

The physics of 1D (one-dimensional) electron and hole systems has been an area of ongoing research interest since conductance quantised in integer multiples of 2*e*^2^/*h* was discovered in short quantum point contacts (QPCs) in GaAs heterostructures^1,2^. The Landauer–Büttiker formalism describes the quantised steps in ballistic 1D conductance by means of transmission probabilities^3^. In QPCs in the quantum limit, many-body interactions lead to an additional anomalous feature below the first conductance plateau at 0.7 × 2*e*^2^/*h*^4,5^. In longer 1D systems, interaction-driven spin–charge separation (where spin and charge excitations travel at different speeds through the 1D constriction) has also been observed^6,7^.

Recently, there has been a resurgence of interest in 1D systems with strong spin–orbit interaction (SOI) due to the potential for engineering non-trivial topological superconductivity. A semiconducting quantum wire with strong SOI can host p-wave superconductivity and Majorana zero-mode states when coupled to a regular s-wave superconductor^8–10^. The system is tuned from the trivial to the topological regime by the application of a magnetic field perpendicular to the effective spin–orbit field **B**_SOI_ in the wire. This mixes the two chiral spin species, opening up a spin gap at **k** = 0. When the Fermi energy *E*_F_ is tuned into this spin gap, the states at *E*_F_ effectively become spinless and Majorana zero modes can form at the ends of the wire.

The key experimental signature of the opening of a spin gap in a quantum wire or point contact with normal contacts is the appearance of a ‘dip’ in conductance on the first 1D subband plateau when a magnetic field is applied parallel to the current direction^11–13^. However, electron–electron interaction effects become strong in the 1D limit, increasing the magnetic susceptibility and spin gap. These interactions cause additional conductance features near 0.7 × 2*e*^2^/*h* that change the spin-gap signatures predicted by single-particle models. Furthermore, unambiguous identification of this spin gap dip is complicated by disorder and finite-length effects in the 1D channel which can also cause dips and oscillations on the first conductance plateau^13–15^. To overcome these complications the 1D system should be free of unwanted disorder and non-adiabatic effects, and the analysis should include many-body interactions.

In this study, we examine the 0.7 anomaly and spin-gap signatures in ultra-low disorder, adiabatic QPCs on GaAs using both electrons (no SOI) and holes (strong SOI). In III-V and group IV semiconductors the conduction band electrons originate from *l* = 0 *s*-shell atomic orbitals, so have weak *l*. *s* SOIs (where $s=\pm \!\frac{1}{2}$ is the electron spin). Valence band holes are formed from *l* = 1 *p*-shell orbitals, so have strong spin–orbit coupling and a total angular momentum $J=L+S=\pm \!\frac{3}{2}$. The 2D quantum well confinement causes a splitting of the ${m}_{J}=\pm \!\frac{1}{2}$ light-hole and ${m}_{J}=\pm \!\frac{3}{2}$ heavy-hole bands at **k** = 0 of order ~10 meV, so that only the heavy hole states are occupied^16^. For both electrons and holes, a magnetic field parallel to the current causes Zeeman splitting of the higher subbands, and a characteristic evolution of the 0.7 anomaly to 0.5 × 2*e*^2^/*h* in magnetic field. However, for holes we find that while the evolution of the conductance is not affected by the strong SOI, the opening of a spin gap shifts the 0.7 anomaly in energy and causes the apparent *g*-factor of the first 1D subband to go to zero. Our results are explained by numerical functional renormalisation group calculations of a tight-binding model that accounts for spin–orbit and strong electron–electron interactions on an equal footing^17^ and we extract a spin–orbit gap *ΔE* ≈ 500 *μ*eV for hole QPCs. Most significantly, we show that rotating the in-plane magnetic field so that it is parallel or perpendicular to the spin–orbit field inside the QPC opens and closes the spin gap, and produces a unique signature of the spin gap in the magnetoconductance.

## Results

**Fig. 1:**
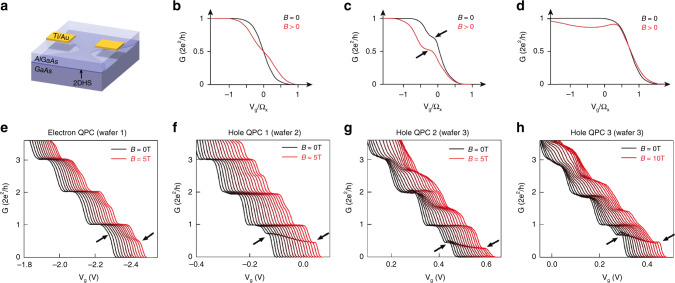
1D conductance in a QPC in magnetic field with spin–orbit and many-body interactions. **a** Schematic of a quantum point contact (QPC), with two gate electrodes biased to define a narrow one-dimensional constriction by locally depleting the 2D electron or hole system in the 2D GaAs/Al_*x*_Ga_1−*x*_As heterostructure. **b** Schematic showing how the conductance *G* of non-interacting electrons in a saddlepoint potential would evolve according to the Landauer–Büttiker model, with a smooth rise from 0 to 2 × 2*e*^2^/*h* as the gate potential *V*_g_ (scaled by the curvature of the 1D constriction Ω_*x*_) is made more negative. Application of an in-plane magnetic field **B** creates an additional step at *G* = 0.5 × 2*e*^2^/*h*. **c** Schematic showing the effect of adding electron–electron interactions, which causes a characteristic shoulder-like anomaly at 0.7 × 2*e*^2^/*h* to appear at **B** = 0. This evolves to 0.5 × 2*e*^2^/*h* in magnetic field, as indicated by the black arrows. **d** Including strong SOI with no electron–electron interactions does not change the situation from the non-SOI case at **B** = 0. For **B** > 0 the conductance rises from 0 to 2*e*^2^/*h*, then dips as the Fermi energy moves through the spin gap in the dispersion relation. **e** Measurements of 1D electrons in a QPC, with a waterfall plot of the conductance showing the evolution of the quantisation from 2*e*^2^/*h* at **B** = 0 (black trace) to *e*^2^/*h* in in-plane magnetic field (**B**∥*I*) up to 5 T (red trace). Traces are offset horizontally for clarity. The 0.7 anomaly is indicated with the black arrow for the **B** = 0 trace, and evolves to 0.5 × 2*e*^2^/*h*. **f**–**h** Measurements of 1D holes in three different QPCs (labelled hole QPCs 1–3) from two different wafers. Waterfall plots show evolution of the conductance quantisation from 2*e*^2^/*h* at **B** = 0 (black trace) to *e*^2^/*h* with in-plane magnetic field up to 10 T (red trace). The field is applied **B**∥*I* (**B**⊥**B**_SOI_) and traces are offset in *V*_g_ for clarity. The 0.7 anomaly is indicated with the black arrow for the **B** = 0 trace, and evolves to 0.5 × 2*e*^2^/*h*.

Figure 1a is a schematic of a typical QPC device (dimensions of all devices are given in Supplementary Table 1). The 2D systems have typical mean free paths of 5 μm for both electrons and holes, and carrier densities of 1.5–2.5 × 10^11^ cm^−2^. Figure. 1b–d shows schematically how the conductance of a QPC with a saddle point potential $V={V}_{0}-\frac{1}{2}m{\omega }_{x}^{2}{x}^{2}+\frac{1}{2}m{\omega }_{y}^{2}{y}^{2}$ depends on the applied magnetic field **B** (the magnetic field axes are scaled with Ω_*x*_, which is set by the curvature of the QPC potential along the direction of current flow), the strength of electron–electron interactions *U*, and spin–orbit interaction *R*. Figure. 1b depicts a conductance plateau at *G* = 2*e*^2^/*h* for *U* = 0 and *R* *=* 0, with an additional step developing at *G* = *e*^2^/*h* with an in-plane magnetic field. Adding electron–electron interactions (Fig. 1c) introduces an additional feature at *G* ~ 0.7 × 2*e*^2^/*h*, which evolves to a plateau at *G* = *e*^2^/*h* with magnetic field. In contrast, when SOIs are added with *U = 0*, the conductance at **B** = 0 is unaffected by the SOI (Fig. 1d). At finite field, the opening of a spin gap leads to a dip in conductance on the 2*e*^2^/*h* plateau.

 Figure 1e–h shows the measured conductance of one electron and three different hole QPCs, fabricated on accumulation mode GaAs/AlGaAs heterostructures. The 1D subbands and 0.7 anomaly show the same behaviour for electrons and holes; at zero magnetic field (leftmost black trace) all QPCs exhibit clean conductance steps, quantised in integer multiples of 2*e*^2^/*h*. The absence of resonance structures is consistent with a low disorder, adiabatic 1D system. Applying an in-plane magnetic field parallel to the current lifts the spin degeneracy and causes additional spin split steps at (*n* + 1/2) × 2*e*^2^/*h*. Whereas the in-plane Zeeman splitting for electrons is isotropic^4^, the strong SOI in hole systems leads to a highly anisotropic Zeeman splitting for the *n* ≥ 2 subbands; the Zeeman splitting for **B**∥*I* is much bigger than that for **B**⊥*I*^18^. This anisotropy has recently been understood as a single particle effect arising from momentum-dependent mixing between light holes and heavy holes^19,20^. The out-of-plane *g*-factor is an order of magnitude larger than the in-plane *g*-factors, so precise alignment of the magnetic field with the 2D hole system (2DHS) is important in order to minimise orbital effects^21^. In this work, the magnetic field is aligned to the 2D system to better than 0.5°. Both electron and hole QPCs also show additional structure below the first subband, indicated by arrows in Fig. 1e–h. In all devices this feature evolves smoothly from 0.7 to 0.5 × 2*e*^2^/*h* with applied magnetic field, a characteristic signature of the 0.7 anomaly. Further evidence that the feature observed in the hole QPCs has the same origin as the 0.7 anomaly in electron QPCs comes from the non-linear differential conductance, which shows the same zero bias peak as observed in electrons^22,23^. Additionally, the reduced conductance in the vicinity of the 0.7 anomaly scales as (1 − **B**^2^), consistent with behaviour of the 0.7 anomaly identified in ref. ^24^ (see Supplementary Information Section 3).

**Fig. 2:**
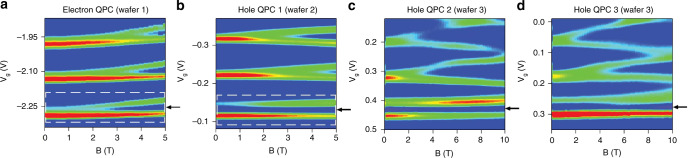
Measured transconductance ∂*G*/∂*V*_g_ of the first three 1D subbands for electrons and holes in QPCs, as a function of energy (gate voltage) and magnetic field. **a** Experimental data from an electron QPC, showing a transconductance colour map of the Zeeman spin splitting of the first three 1D subbands as a function of gate voltage *V*_g_ and magnetic field **B**. Dark-blue regions correspond to conductance plateaus, and the green to red regions correspond to conductance risers (which mark the subband edges). The dashed white boxes in (**a**) and (**b**) mark the first subband and are examined in greater detail in Fig. 3. Each subband splits linearly in magnetic field, including the 0.7 anomaly (indicated by the black arrow). **b**–**d** Experimental transconductance colour maps of the Zeeman spin splitting of the first three 1D hole subbands for hole QPCs 1–3. In all the cases, subbands 2 and 3 spin-split linearly in magnetic field, whereas the first hole subband is only weakly affected by the magnetic field.

In contrast to the linear response conductance, which is the same for electrons and holes, the strong SOI fundamentally alters the energy-dependent behaviour of the first 1D subband in magnetic field, as shown in Fig. 2. The transconductance *dG*/*dV*_g_ probes the local density of states in the QPC, and is routinely used to map the 1D subband edges as a function of energy (gate voltage). Figure. 2a–f shows the transconductance colour maps, plotted against gate voltage and magnetic field for the same four QPC devices in Fig. 1e–h. In Fig. 2a, all the first three 1D electron subbands spin-split linearly in magnetic field, with no qualitative difference between the subbands. The arrow indicates the position of the 0.7 anomaly. In contrast, the 1D hole systems in Fig. 2b–d show a linear spin-splitting of the second and third subbands, while the splitting of the first subband is almost unaffected by the magnetic field. We note that the conductance behaviour in Fig. 1f–h and transconductance behaviour in Fig. 2b–d is reproduced for a further three hole QPCs in Supplementary Information Section 4, and has also been observed in previous studies, although it has remained unexplained^21,25,26^.

**Fig. 3:**
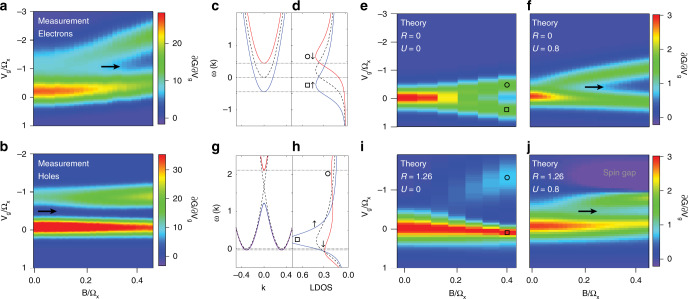
Comparison of measured 1D transconductance to tight-binding numerical calculations. **a** Close up of the measured transconductance of the first 1D subband in the electron QPC (the region in the white dashed box in Fig. 2a), showing the first 1D electron subband spin-splits in magnetic field. Gate voltage *V*_g_ and magnetic field **B** axes are converted to energy and scaled with the QPC constriction curvature parameter Ω_*x*_ for comparison with theory. **b** Close up of the measured transconductance from hole QPC 1 (the region in the white dashed box in Fig. 2b), showing the transconductance of the first 1D hole subband is clearly different to electron QPCs, and does not split in magnetic field. **c** Calculated dispersion relation of the first spin-resolved subband (spin-up shown in blue, spin-down in red) in **B** > 0. The vertical axis is energy *ω* and the horizontal axis is wavevector **k**. The **B** = 0 dispersion relation is indicated by the black dotted line. **d** The local density of states (LDOS) calculated from the dispersion relation shown in (**c**), plotted against energy *E*. Again the LDOS is shown for each resolved spin-species in blue and red (indicated by the up and down arrows), and the black dotted line is the LDOS in zero magnetic field. **e** Calculated transconductance colour map of the first 1D subband with no Rashba SOI (*R* = 0) and no on-site Coulomb interactions (*U* = 0). The circle and square markers correspond to the two spin-resolved peaks in the LDOS in (**d**). **f** Calculated transconductance colour map of the first 1D subband with no Rashba SOI and finite on-site Coulomb interactions *U* = 0.8. The 0.7 anomaly is indicated by the black arrow. **g** Calculated dispersion relation of the first subband with spin-mixing in magnetic field. The pure spin-states are indicated by the red and blue regions in the dispersion relation at **k** = 0. The spin-mixed states are indicated by the purple regions away from **k** = 0. **h** The LDOS for the dispersion model shown in (**g**). For **B** > 0, the spin-up species in blue forms a single large peak, while the spin-down species in red forms two smaller peaks, with the peak at low energy emerging because of spin-mixing. **i** Calculated transconductance colour map of the first 1D subband with Rashba SOI *R* = 1.26 and zero on-site Coulomb interactions *U* = 0. Circle and square markers correspond to the LDOS peaks indicated by the same markers in panel (**h**). **j** Calculated transconductance colour map of the first 1D subband with Rashba SOI magnitude *R* = 1.26.

In Fig. 3a we zoom in on the first 1D electron subband from Fig. 2a, and compare it directly to Fig. 3b where we zoom in on the first 1D hole subband of hole QPC 1 from Fig. 2b. In both Fig. 3a and b, the gate voltage and magnetic field axes have been scaled with Ω_*x*_ to allow comparison with theory. Close up, the differences between electrons and holes become very clear; the first 1D electron subband has a weakly resolved 0.7 anomaly structure at **B** = 0 that splits in magnetic field. In contrast, the first 1D hole subband has a strongly resolved 0.7 anomaly structure at **B** = 0 that does not broaden in energy as magnetic field increases, along with the two transconductance peaks that do not split.

The apparent suppression of the spin-splitting in the first 1D hole subband is unexpected, since the magnetic field strongly affects the conductance of the first 1D hole subband, as shown in Fig. 1f–h, indicating that the *g*-factor cannot be zero. We also cannot attribute this behaviour to peculiarities of the in-plane *g*-factor anisotropy; even if **B** is applied out-of-plane, where the *g*-factor is an order of magnitude larger than the in-plane *g*-factors, the first subband shows no spin-splitting of transconductance up to 0.9 T, whereas the higher subbands have already entered the quantum Hall regime (see Supplementary Information Section 3).

To understand the difference between electron and hole systems in the 1D limit we study an infinite tight-binding chain at zero temperature in the presence of SOIs and an external magnetic field^17^. The first subband of the QPC is modelled as a smooth potential barrier, which is non-zero only in a finite region, separating two semi-infinite leads. Electron–electron interactions are also present only in the central QPC region of the system. Without electron–electron interactions this model is exactly solvable, while the interacting model can be studied using functional renormlisation group (fRG) theory. This model has been used for electron QPCs to reproduce the observed conductance of the 0.7 anomaly, as well as for reproducing the shot noise and compressibility, due to increased electron–electron interactions, inelastic scattering, and increased magnetic susceptibility^24^. This model has been extended to ‘heavy’ electrons with the inclusion of a Rashba SOI term to make predictions for the 0.7 anomaly in hole QPCs.^17^.

Assuming (without loss of generality) that for carriers travelling in the *x*-direction the effective spin–orbit field **B**_SOI_ is parallel to the *y*-axis, the Rashba energy contribution equals −*ασ*_*y*_**k**, where **k** is the momentum of the electron, *α* characterises the strength of the SOI, and *σ*_*y*_ is a Pauli matrix. Without an external magnetic field, this contribution results in a negative energy offset in the dispersion relation of magnitude *ΔE*_SOI_ = *α*^2^m*/2*ℏ*^2^, where m* is the effective mass of the charge carriers. We parameterise the Rashba SOI by the dimensionless number ${\mathcal{R}}$,

1 $${\mathcal{R}}=\sqrt{\frac{\Delta {E}_{\mathrm{SOI}}}{{\Omega }_{x}}}=\frac{{\alpha }_{R}}{\hslash }\sqrt{\frac{{m}^{* }}{2{\Omega }_{x}}},$$

Further details of the model are given in Supplementary Information Section 1 and in refs. ^17,24^. We note that a strong SOI is a necessary, but not sufficient, condition to observe a spin gap in the conductance; the simple picture of the spin gap causing a conductance dip from 2*e*^2^/*h* to *e*^2^/*h* with an applied field assumes an infinitely long, translationally invariant quantum wire. A finite-length system will, in practice, exhibit a much weaker conductance dip due to lifetime broadening of the 1D eigenstates in the wire^14,17^. Physical insight into the effective strength of the SOI in the 1D channel, ${\mathcal{R}}$, can be obtained by re-expressing Eq. (1) as

2 $${\mathcal{R}}\propto \sqrt{\frac{\Delta {E}_{\mathrm{SOI}}}{h/{\tau }_{\mathrm{transit}}}}$$

where *ΔE*_SOI_ is the size of the spin gap, and *h*/*τ*_transit_ is the energy uncertainty arising from the finite lifetime of ballistic charge carriers moving through the finite length 1D constriction. If this energy broadening is larger than *ΔE*_SOI_, i.e. ${\mathcal{R}}\,<\,1$, then the spin gap cannot be resolved. Even if ${\mathcal{R}}\,> \,1$ the spin gap may only cause a small dip in the conductance.

We start our discussion with the ‘simple’ case of the first 1D electron subband, where there is no SOI, in the presence of a magnetic field **B**. The 1D subband dispersion for non-interacting electrons is parabolic and spin-resolved in energy due to Zeeman spin-splitting, as shown in Fig. 3c. The local 1D density of states (LDOS) at the top of the barrier is shown for the two spin species in Fig. 3d. The open circle and open square indicate the spin-split peaks in the LDOS. The transconductance is a direct probe of the LDOS; the calculated transconductance colour map in Fig. 3e shows a linear splitting of the transconductance peaks with field **B**. The absence of electron–electron interactions means there is no 0.7 anomaly. In Fig. 3f we include a finite on-site Coulomb interaction *U* = 0.8. This causes an enhanced and asymmetric splitting of the transconductance peaks, consistent with an enhanced spin susceptibility, and in good agreement with measurements of the 1D electron device in an in-plane magnetic field shown in Fig. 3a. The on-site Coulomb interaction also gives rise to the 0.7 anomaly at finite field.

In Fig. 3g onward, we now include a strong SOI where the Rashba SOI coefficient *α*_*R*_ = 0.3, which is equivalent to ${\mathcal{R}}=1.26$, consistent with the estimated strength of the SOI in the 2D hole system and the confining potential in the 1D QPC (see Supplementary Information Section 3). At zero magnetic field the 1D subbands are separated in momentum by ±**k**_SOI_ due to the Rashba interaction. Applying a magnetic field parallel to the current causes spin-mixing and the opening of a spin gap at **k** = 0 in the 1D hole dispersion, as shown in Fig. 3g. This spin-mixing causes a strong enhancement of the low energy peak in the spin-‘up’ LDOS (□), while the spin-‘down’ LDOS splits into two smaller peaks: one below the enhanced spin-‘up’ peak, and one higher in energy (∘) (Fig. 3h). This splitting of the spin-‘down’ LDOS peak, with the resultant suppression in the LDOS in the vicinity of *ω*(**k**) = 1.2, corresponds to the spin gap in the dispersion relation in Fig. 3g. The higher energy peak in the LDOS (∘) marks the energy at which the spin gap closes.

Spin-mixing of the Rashba split bands means that the energies of the low-energy spin-‘down’ peak and the enhanced spin-‘up’ peak (□) are only very weakly dependent on magnetic field; they are effectively ‘pinned’ with respect to energy. This pinning is evident when we plot the transconductance in Fig. 3i, where there is a strong, single first subband peak (□) that hardly moves in energy. The weaker peak that is higher in energy (∘) in transconductance emerges in finite **B** and then moves rapidly up in energy as **B** increases. Again, the absence of electron–electron interactions means there is no 0.7 anomaly.

In Fig. 3j we turn on the Coulomb interactions, which significantly changes the behaviour of the transconductance. The enhanced spin-‘up’ peak and the low-energy spin-‘down’ peak that formed one large peak in Fig. 3i now form two transconductance peaks that run parallel to each other in magnetic field, with the 0.7 anomaly in between (marked by the black arrow) that does not shift in gate voltage (energy). The enhanced magnetic susceptibility strengthens the spin gap, making it visible at lower magnetic field, as indicated by the purple region in the top right of Fig. 3j. The key features of spin–orbit and electron–electron interactions in combination are the pinning of the two transconductance peaks, and the formation of a spin gap feature in the transconductance. These features are distinct from the observed transconductance in electron devices where SOI is weak or close to zero.

The pinning of the transconductance peaks and 0.7 anomaly produced in calculations is in very close agreement with the observed behaviour of the first 1D hole subband transconductance in Fig. 3b, and is compelling evidence for the SOI in our hole QPCs being sufficiently strong to open a spin gap. We note that that although the *T* = 0 fRG calculations are unable to fully reproduce the *T* > 0 experimental behaviour of the 0.7 anomaly at **B** = 0 (see refs. ^17,24^ and Supplementary Information Section 1), we do expect and observe good agreement at finite **B** where both the 0.7 anomaly and spin gap are present. The absence of an observable spin-gap signature in the conductance across all six of the hole QPCs presented here and in Supplementary Information Section 4 indicates that simply applying a magnetic field along a 1D system may not be a reliable method of detecting spin gap physics. A new spin-gap signature could therefore be a valuable tool for studying spin physics in 1D systems.

**Fig. 4:**
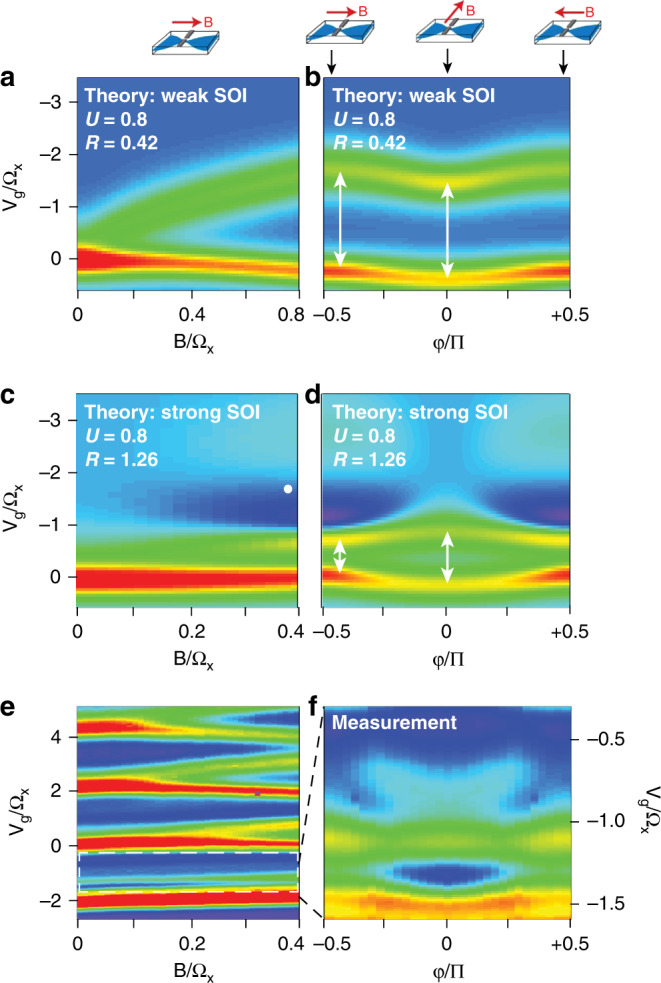
Transconductance of a QPC in magnetic field with spin–orbit and many-body interactions. **a** Calculated transconductance of first 1D subband with small Rashba SOI ${\mathcal{R}}=0.42$ in increasing magnetic field up to **B**/Ω_*x*_ = 0.8. **b** Calculated transconductance of the first 1D subband with small ${\mathcal{R}}=0.42$ as a function of magnetic field angle *φ* where the magnitude of the magnetic field is fixed at **B**/Ω_*x*_ = 0.8. The width of the energy gap between the two transconductance peaks is indicated by the white arrows. **c** Calculated transconductance of first 1D subband with strong ${\mathcal{R}}=1.26$ (abbreviated from Fig. 3j) in increasing magnetic field up to **B**/Ω_*x*_ = 0.4. The white dot indicates the region corresponding to the spin gap conductance minima in the vicinity of **B**/Ω_*x*_ = 0.4. **d** Calculated transconductance of the first 1D subband with small ${\mathcal{R}}=1.26$ as a function of magnetic field angle *φ* where the magnitude of the magnetic field is fixed at **B**/Ω_*x*_ = 0.4. The width of the energy gap between the two transconductance peaks is indicated again by white arrows. **e** Measured transconductance of first 1D hole subband (abbreviated from Fig. 3b) in increasing magnetic field up to **B** = 4 T. **f** Measured transconductance of the first 1D hole subband as a function of magnetic field angle *φ* where the magnitude of the magnetic field is fixed at **B** = 4 T.

If the pinning of the first two transconductance peaks is related to strong SOIs and the opening of a spin gap, it should be extremely sensitive to the orientation of the in-plane magnetic field, since the spin gap will close if **B**∥**B**_SOI_. Figure. 4 shows the calculated and measured angular dependence of the transconductance. We start by considering ${\mathcal{R}}=0.42$ (*α*_*R*_ = 0.1), for which we do not expect to observe spin gap physics (due to lifetime broadening). Figure. 4a shows the first 1D subband transconductance peak splitting as the magnetic field applied parallel to the current direction is increased up to ∣**B**∣ = 0.2Ω_*x*_. Figure. 4b shows the evolution of these two transconductance peaks as a function of in-plane magnetic field angle *φ*, for fixed ∣**B**∣. Despite the presence of the SOI, the energy gap between the two peaks remains constant as a function of magnetic field orientation, as indicated by the white arrows, although both peaks shift slightly down in energy around *φ* = 0 (where ${\bf{B}}\perp \overrightarrow{I}$).

Increasing the strength of the SOI to ${\mathcal{R}}=1.26$ causes the picture to change dramatically, as shown in Fig. 4c, d. The transconductance peak at **B** = 0 no longer splits with increasing **B**; instead the peak stays almost fixed at *V*_g_ = 0 in magnetic field, with the second peak emerging at *V*_g_ = −0.8 at higher fields. For **B** > 0.1Ω_*x*_ a weak dip in the conductance around *V*_g_ = −2 due to spin gap opening causes additional transition from dark blue to light blue as *V*_g_ becomes more negative, as highlighted by the white spot on the figure. Rotating the magnetic field orientation changes both the splitting of the first two transconductance peaks and the spin gap, as shown in Fig. 4d. The two blue ‘wing-like’ structures associated with the spin gap in the range −1 > *V*_g_/Ω_*x*_ > −3.5 disappear as ∣*φ*/∣*π* → 0, where ${\bf{B}}\perp \overrightarrow{I}$ and the spin gap closes. Lower in energy, the two transconductance peaks no longer have a fixed separation: the 0.7 anomaly is ‘squashed’ by the opening spin gap away from ∣*φ*∣/*π* = 0, indicated by the short arrow. As the field is rotated towards ∣*φ*∣/*π* = 0, the 0.7 anomaly broadens, indicated by the longer arrow. The light blue structures at high energy, and the narrowing of the 0.7 structure away from ∣*φ*∣/*π* = 0, provide unique signatures of the spin gap.

Figure 4e shows the first 1D subband transconductance peak splitting as the magnetic field applied parallel to the current direction is increased up to ∣**B**∣ = 4 T. The transconductance peaks from the *n* ≥ 2 subbands show a characteristic Zeeman splitting, while the ones associated with the first 1D subband are almost unaffected by **B**. However, changing the magnetic field orientation at fixed ∣**B**∣ = 4 T (the out of plane component of the magnetic field is always less than 4 mT). has a clear effect on the first 1D subband, as shown in Fig. 4f (note that Fig. 4f is taken over the field orientation range of ∣*φ*∣/π = 0.5 or 90^∘^, and is presented as a mirror image for easy comparison with theory. For the full data set taken over 240^∘^ including higher subbands and further analysis of the 1D subband spacings, see Supplementary Information Section 6.). The data show the same squashing of the 0.7 anomaly away from ∣*φ*∣/*π* = 0 as in Fig. 4d. More significantly, there are also ‘wing-like’ structures that emerge in the range −1 > *V*_g_/Ω_*x*_ > −1.5 as ∣*φ*∣ increases. We note that in the theoretical model these ‘wing-like’ structures do not occur for ${\mathcal{R}}\,<\,1$; they only occur for sufficiently strong SOI that the spin gap is larger than the lifetime broadening.

The main discrepancy between theory in Fig. 4d and experiment in Fig. 4f is due to the impact of the second 1D hole subband, which is not considered in our purely 1D model. The ‘wing-like’ structures associated with the spin gap are more prominent in the calculations than in the experiments, where they do not extend all the way to the edge of the figure but vanish as ∣*φ*∣/*π* → ±0.5 (${\bf{B}}\parallel \overrightarrow{I}$). The absence of the spin gap structure when (${\bf{B}}\parallel \overrightarrow{I}$) is consistent with the measurement shown in Fig. 3b, where a spin gap structure is not observed despite the SOI being sufficiently strong to cause the transconductance peaks to split and run parallel to each other as magnetic field is increased. We attribute the absence of an observable spin gap structure at ${\bf{B}}\parallel \overrightarrow{I}$ to the presence of the second, spin-split 1D subband moving down in energy. The proximity of the second 1D subband to the first 1D subband in energy is shown here to be a key factor in the ‘visibility’ of any spin-gap signature, and may in part explain the ongoing difficulty in unambiguously detecting spin-gap signatures in QPCs. This problem may be exacerbated in higher 1D subbands where the 1D subband spacing is much smaller than the first and second 1D subband spacing, and spin-gap signatures have been predicted to occur but have not been observed^27^. Further analysis and discussion of the higher 1D hole subbands is provided in Supplementary Information Section 6.

## Discussion

In experimental systems strong electron–electron interactions are always present in the 1D limit, and so must be considered on an equal footing with the SOI. Our measurements of the first 1D subband in QPCs with and without strong SOI demonstrate that the SOI fundamentally alters the behaviour of the first 1D hole subband compared to the first 1D electron subband. The experimental data and the modelling both show that the magnetic field evolution of the transconductance is a much more sensitive probe of the spin gap than the conductance. Although the model does not contain some of the more complex spin-physics of holes, it nevertheless reproduces the key experimental observations: (i) despite the magnetic field causing the 0.7 anomaly to evolve towards 0.5 × 2*e*^2^/*h*, the associated transconductance peaks remain pinned in energy and hardly change as **B** is increased, and (ii) rotating the magnetic field causes characteristic features to appear in the transconductance.

By comparing the experimental data of Figs. 2–4 with theory we can obtain an estimate of the spin–orbit gap. We calculated the transconductance for a range of SOI values $0\le {\mathcal{R}}\le 1.26$, electron–electron interaction strengths 0 ≤ *U* ≤ 0.8, and magnetic fields 0 ≤ **B** ≤ 0.88Ω_*x*_ and then compared them to the measured transconductance. We found ${\mathcal{R}}=1.26$ and *U* = 0.8 to be in closest agreement with experiment in Fig. 3j and **B** = 0.4Ω_*x*_ in Fig. 4d. Using ${\mathcal{R}}=1.26$ and Eq. (1), we estimate the size of the spin gap in the device in Fig. 3b to be *ΔE* = 550 ± 100 *μ*eV. This is consistent with the value expected from independent measurements of the Rashba splitting in the 2D hole system (see Supplementary Information Section 3).

Finally, we remark on the impact of this work on topological superconductivity and Majorana physics in 1D systems. To enter the topological regime strong SOI, low disorder and superconducting contacts are prerequisites. The wing-like structure shown in Fig. 4 is a universal and unambiguous signature of the spin gap, and can be used to tune the system into the topological regime. Our work also shows that the effective strength of the SOI in the 1D system should be large (the 1D system should be as long as possible while maintaining ballistic transport, to maximise ${\mathcal{R}}$), and the 1D subband spacing should be maximised. With the recent demonstration of superconducting contacts to ultra-low disorder 2D electron systems in GaAs/AlGaAs heterostructures, and to high mobility holes in Ge quantum wells^28,29^, this work shows a route to scalable topological superconducting circuits.

## Methods

### Experimental set-up

All devices for these experiments were fabricated on undoped accumulation mode (100) GaAs/Al_*x*_Ga_1−*x*_As heterostructures, using standard electron beam lithography techniques to define the QPCs. Details of all the wafers used and dimensions of the QPCs, are given in Supplementary Information Section 4. Measurements were performed in dilution refrigerators with base temperatures below 40 mK, using standard low-frequency ac lock-in techniques with an applied excitation voltage of *V*_sd_ = 50 to 100  μV, where typically more than half of *V*_sd_ is dissipated across the 2DEG/2DHG, ohmic contacts and cold filters. Typical electron and hole densities were from 1.0 to 2.5 × 10^11^ cm^−2^, with electron and hole mobilities above 1 × 10cm^2^V^−1^s^−1^.

## Supplementary information

Supplementary Information

## Data Availability

The data that support the findings of this study are available from the corresponding author upon reasonable request.
