# Supplementary material for: New signatures of the spin gap in quantum point contacts
Source: Nat Commun. 2021 Jan 4;12:5. doi: 10.1038/s41467-020-19895-3 (PMC7782751; doi:10.1038/s41467-020-19895-3)
Supplement: Supplementary file 1 — Supplementary Information [file 41467_2020_19895_MOESM1_ESM.pdf]

Supplementary Information: New signatures of the spin gap in quantum point contacts

K. L. Hudson[1,2], A. Srinivasan[1,2], O. Goulko[3], J. Adam[1], Q. Wang[1,2], L. A. Yeoh[1], O. Klochan[1,2], I. Farrer[4], D. A. Ritchie[5], A. Ludwig[6], A.D. Wieck[6], J. von Delft[7] & A. R. Hamilton[1,2*]

[1] *School of Physics, University of New South Wales, Sydney, NSW, 2052, Australia.*

[2] *ARC Centre of Excellence in Future Low-Energy Electronics Technologies, University of New South Wales, Sydney, NSW, 2052, Australia.*

[3] *Department of Physics, University of Massachusetts, Boston, MA, 02125, USA.*

[4] *Cavendish Laboratory, University of Cambridge, Madingley Road, Cambridge, United Kingdom.*

[5] *Department of Electronic and Electrical Engineering, University of Sheffield, Sheffield, United Kingdom.*

[6] *Angewandte Festkörperphysik, Ruhr-Universität Bochum, D-44780 Bochum, Germany*

[7] *Arnold Sommerfeld center for Theoretical Physics, Ludwig-Maximilians Universität, München, Theresienstrasse 37, D-80333, München, Germany.*

⋆ *e-mail: alex.hamilton@unsw.edu.au*

1 Notes on theory **S-3**

2 Subband properties and calculation of $\mathcal{R}$ for QPCs **S-6**

3 Further evidence of dependence of first subband with spin-orbit interaction in magnetic field **S-11**

4 Fabrication and measurement methods **S-23**

1 Notes on theory

Our theoretical description [1] is based on the following infinite tight-binding chain Hamiltonian,

$$
\begin{aligned}
H \;=\; & \sum_{j,\sigma,\sigma'} d^{\dagger}_{j\sigma}\left[(V_j+2\tau)\delta_{\sigma\sigma'}-\frac{1}{2}(\boldsymbol{\sigma}\cdot\boldsymbol{B})_{\sigma\sigma'}\right]d_{j\sigma'} \\
& +\sum_{j,\sigma,\sigma'}\left[d^{\dagger}_{j+1\sigma}\left(-\tau_0\delta_{\sigma\sigma'}+\frac{i\alpha}{2}(\sigma_y)_{\sigma\sigma'}\right)d_{j\sigma'}+\text{h.c.}\right] \\
& +\sum_{j} U_j d^{\dagger}_{j\uparrow}d_{j\uparrow}d^{\dagger}_{j\downarrow}d_{j\downarrow},
\end{aligned}
\tag{1}
$$

where $d_{j\sigma}$ and $d^{\dagger}_{j\sigma}$ annihilate and create an electron with spin $\sigma \in \{\uparrow,\downarrow\}$ at site j, respectively, and $\boldsymbol{\sigma} = (\sigma_x,\sigma_y,\sigma_z)$ is a vector of Pauli matrices. The external magnetic field $\boldsymbol{B}$ and spin orbit parameter α are constant throughout the chain. The effective mass of the charge carrier is $m = \hbar^2/2\tau a^2$ with $\tau = \sqrt{\tau_0^2+\alpha^2}$, where τ_0 is the hopping between sites in the discrete model and a is the spacing between sites. We keep τ fixed when varying α, in order to ensure that the effective mass in the discrete model matches the physical effective mass. This is equivalent to matching the bandwidth.

In our calculations the QPC region consists of 101 sites centered around $j = 0$ and thus has overall length $L = 2Na$ with $N = 50$. Sites $j < -N$ and $j > N$ represent two leads with bandwidth 4τ. The QPC barrier potential,

$$V_j = V(ja) = (V_{\mathrm{g}}+\mu)\exp\left[-\frac{(2ja/L)^2}{1-(2ja/L)^2}\right], \tag{2}$$

and the (on-site) electron-electron interaction,

$$U_j = U(ja) = U\exp\left[-\frac{(2ja/L)^6}{1-(2ja/L)^2}\right], \tag{3}$$

are nonzero only in the QPC region. Both the barrier potential and interaction are symmetric around the center and vanish smoothly at the boundary of the QPC region. The barrier potential is quadratic around the central site $j = 0$, representing the lowest QPC subband. The barrier height V_{g}, measured w.r.t. the chemical potential $\mu = 2\tau$, mimics the role of the gate-voltage. If V_{g} is swept downwards through zero, the linear conductance g increases from 0 to 1.

In the main paper we discuss results for the following physical quantities at zero temperature: the local density of states (LDOS), $\mathcal{A}^{\sigma}_{j=0}$, at the central site of the system (with $U = 0$) and the linear conductance, g. The former is given by

$$\mathcal{A}^{\sigma}_{j}(\omega) = -\mathrm{Im}\mathcal{G}^{\sigma\sigma}_{jj}(\omega)/\pi a, \tag{4}$$

where $\mathcal{G}^{\sigma\sigma'}_{jj'}$ is the retarded propagator from site j' with spin σ' to site j with spin σ. Due to SOI, $\mathcal{G}^{\sigma\sigma'}_{jj}$ is not spin-diagonal, but at $j = 0$ its off-diagonal elements are negligible compared to the diagonal ones. The linear conductance can be calculated via [2]

$$g = g_1 + g_2 \propto \mathrm{Tr}(t^{\dagger}t) = \sum \mathrm{eig}(t^{\dagger}t), \tag{5}$$

where $t^{\sigma\sigma'} = \mathcal{G}^{\sigma\sigma'}_{-N,N}(\mu)$ is the transmission matrix of the QPC. The eigenvalues of $t^{\dagger}t$, which yield the conductance, are independent of N.

The non-interacting system ($U = 0$) can be solved exactly. In the presence of electron-electron interactions, we calculate the conductance at zero temperature with the functional Renormalization Group technique in the one-particle irreducible version [3–7] using the coupled ladder approximation, which was presented in Ref [8] for a model without SOI.

Note on $T = 0$ calculations and finite T experiments

The $T = 0$ fRG theory captures the behaviour of the 0.7 anomaly at finite magnetic field, in good agreement with experimental data. This is the regime in which the key physics of spin gap is observed, and is the regime we concentrate on in this work. The agreement between the calculations and measurement is less good at low B, where finite T effects become more significant. Here the $T = 0$ theory appears to show a suppression of the 0.7 anomaly at low B, which does not occur in experiment. This suppression is a limitation of the fRG technique used to solve the 1D model; finite T calculations using second order perturbation theory do not show a suppression of the 0.7 anomaly at $T > 0, B = 0$, but do not fully capture the electron-electron interactions, as discussed in Ref. [9].

2 Subband properties and calculation of $\mathcal{R}$ for QPCs

Summary of QPC parameters

Here we summarise the gate dimensions, charge density, spin polarisation and provide and estimate of the magnitude of $\mathcal{R}$ for each QPC device in Supplementary Tables 1 and 2. The method for calculating these values including is provided below, along with discussion about the challenges of obtaining an accurate value of $\mathcal{R}$.

Device	Wafer no.	2DEG/2DHG depth (nm)	$l \times w$ (nm)	$n_{2D}\,(10^{11}\mathrm{cm}^{-2})$	$\Delta p/p$	$\mathcal{R}$	ΔE_{SOI} (μeV)
Electron QPC	1 [W639]	160	60×350	1.5	0	0	0
Hole QPC 1	2 [W713]	85	300×300	1.2	0.10	$0.7^{+0.41}_{-0.34}$	580
Hole QPC 2	3 [W917]	60	100×300	2.5	0.46	$1.6^{+0.36}_{-0.19}$	450
Hole QPC 3	3 [W917]	60	100×300	2.5	0.46	$1.6^{+0.36}_{-0.19}$	480
Hole QPC 4	1 [W639]	160	200×800	2.5	0.29	$1.8^{+0.30}_{-0.35}$	250
Hole QPC 5	4 [B13180]†	200	400×400	1.5			
Hole QPC 6	5 [W918]	60	350×350	2.1	0.40	$2.5^{+0.18}_{-0.03}$	960

Supplementary Table 1. Summary of measured spin populations and spin-orbit parameter $\mathcal{R}$ calculated from $\Delta p/p$ and l for each device. † Characterisation of Rashba SOI via Shubnikov-de Haas oscillations were not performed for this wafer. No value for $\Delta p/p$ or $\mathcal{R}$ available.

Device	Wafer no.	2DEG/2DHG depth (nm)	$l \times w$(nm)	Ω_y(μeV)	Ω_x(μeV)
Electron QPC	1 [W639]	160	60×350	1900	600
Hole QPC 1	2 [W713]	85	300×300	490	150
Hole QPC 2	3 [W917]	85	100×300	475	180
Hole QPC 3	3 [W917]	85	100×300	500	190
Hole QPC 4	1 [W639]	160	200×800	290	75
Hole QPC 5	4 [B13180]	200	400×400	240	110
Hole QPC 6	5 [W918]	60	350×350	520	150

Supplementary Table 2. Summary of subband spacings Ω_y and Ω_x for each device.

Calculating $\mathcal{R}$ via $\Delta p/p$ and QPC length l

The value of $\mathcal{R}$ can be obtained from the measured strength of the spin-orbit interaction in the 2DEG/2DHG system and the length of the QPC. The Rashba interaction in 2D systems derived from the Luttinger Hamiltonian is:

$$H = \frac{1}{2m_e}\left(\left(\gamma_1 + \frac{5}{2}\gamma_2\right)p^2 - 2\gamma_2(p \cdot S)^2\right) \tag{6}$$

The 2D potential is applied to restrict motion in the z-direction. At experimental densities, we have $\langle p_z^2 \rangle \gg p_F^2 = p_x^2 + p_y^2$, so that the dominant terms in (1) are proportional to $p_z^2 S_z^2$ and are diagonal in a basis of states with definite S_z. States with $S_z = \pm\frac{3}{2}$ (heavy holes) are split in energy from

states with $S_z = \pm\frac{1}{2}$ (light holes). The light holes are higher in energy by a splitting equal to

$$\Delta_{HH-LH} = -\frac{2\gamma_2\langle p_z^2\rangle}{m_e} \approx 10\,\mathrm{meV}, \tag{7}$$

so that the low energy sector consists of a doublet of heavy holes.

The remaining terms containing S_x,S_y introduce mixing between heavy and light hole states. Accounting for a correction in third order perturbation theory due to the terms

$$-\frac{\gamma}{m_e}((p_xS_x + p_yS_y)^2 + \{p_xS_x + p_yS_y\, p_zS_z\}) \tag{8}$$

we find that the interaction is of the form

$$H_R = \frac{ia}{2}(p_+^3\sigma_- - p_-^3\sigma_+) \tag{9}$$

where a is a numerical coefficient depending on the shape of the well. Due to parity selection rules, $a = 0$ for a symmetric well.

The Rashba interaction splits the 2D bands. The dispersion has the form

$$\varepsilon_p^\pm = \frac{p^2}{2m} \pm \alpha p^3. \tag{10}$$

Magnetotransport experiments in 2D hole systems show two Fermi surfaces with carrier densities p_+ and p_- that are typically different by a factor of two, although this factor may be as large as twenty. The strength of the Rashba interaction may be extracted in a straightforward manner. Introducing the parameter

$$p = \sqrt{2mE_F} \tag{11}$$

we can characterise the spin-orbit interaction in terms of the dimensionless constant

$$\frac{\alpha p^3}{E_F} = 2m\alpha p \approx \frac{\alpha p_+^3}{E_F} \approx \frac{\alpha p_-^3}{E_F} \tag{12}$$

This can be solved given knowledge of p_+, p_-

$$E_F = \frac{p_+^2}{2m} - \alpha p_+^3 = \frac{p_-^2}{2m} + \alpha p_-^3 \to 2m\alpha p = p\frac{p_+^2 - p_-^2}{p_-^3 + p_+^3} \tag{13}$$

For QPC 4 (a typical experimental situation), the densities p_- and p_+ are in the ratio $0.29 : 0.71$. Writing

$$p_+^2 = 0.71(2p^2), \;\; p_-^2 = 0.29(2p^2) \tag{14}$$

we can substitute into (8) and find

$$2m\alpha p = \frac{(2p^2)\cdot p(0.71 - 0.29)}{(2p^2)^{\frac{3}{2}}(0.71^{\frac{3}{2}} + 0.29^{\frac{3}{2}})} = 0.28 \tag{15}$$

The large value of this dimensionless size of the spin-orbit interaction reflects the fact that, in inversion asymmetric 2D hole systems, the Rashba interaction is a considerable proportion of the kinetic energy.

In a QPC, an additional harmonic potential is applied to the confine the electrons to a 1D channel. Therefore momentum along the y-direction is quantized. We can make the substitution $\langle p_y^2 \rangle \to 2mE_F$, $\langle p_y \rangle = 0$. Then expanding the 2D Hamiltonian we obtain

$$H_R \to -3ap_y^2 p_x \sigma_y + ap_x^3 \sigma_y \tag{16}$$

Near the top of the barrier, $p_x \to 0$ and the first term dominates. We may write it in the form

$$H_R \to -\alpha p_x \sigma_y, \;\; a = 3\alpha p_y^2 = 6m\alpha E_F \tag{17}$$

The dimensionless parameter R is defined to be the ratio of α to the velocity scale determined by the shape of the parabolic barrier inside the channel, $U(x) \approx U_0 - \frac{m\Omega_x^2 x^2}{2}$

$$\mathcal{R} = \frac{\alpha}{v} = \alpha\sqrt{\frac{m}{\Omega_x}} \tag{18}$$

From the expression for the Fermi energy E_F inside the parabolic potential

$$E_F = \frac{m\Omega_x^2 l^2}{8} \rightarrow \Omega_x = \sqrt{\frac{8E_F}{ml^2}} \tag{19}$$

we can express $\mathcal{R}$ in terms of the 1D channel length

$$\mathcal{R}^2 = \frac{m\alpha^2}{\Omega_x} = \frac{m\alpha^2}{2}\sqrt{\frac{ml^2}{2E_F}} = \frac{(m\alpha)^2}{2}\sqrt{\frac{l^2}{2mE_F}} = \frac{lp}{2}\left(\frac{m\alpha}{p}\right)^2 \tag{20}$$

Using a density of $2.5 \times 10^{11}\mathrm{cm}^{-2}$ and length $l = 300\,\mathrm{nm}$ as typical values, and the value of $2m\alpha p$ found in equation (10), we find

$$\frac{m\alpha}{p} = 3map = \frac{3}{2}(2m\alpha p) = 0.42 \tag{21}$$

This yields a final value of $\mathcal{R}^2 = 3.3 \rightarrow \mathcal{R} \approx 1.8$.

The main limitation of using equation (20) to estimate $\mathcal{R}$ is the uncertainty in the true QPC length felt by the first harmonic, which will likely be longer than the lithographic gate length. The QPC length felt by the first 1D subband is determined by the electrostatic profile of the surface gates when the QPC is being squeezed, which is determined by the dimensions of the surface gates and the depth of the 2DHG. The method above provides a reasonable estimation of $\mathcal{R}$. However we argue that the form of the first 1D subband in magnetic field (i.e., the absence of Zeeman spin-splitting) is the most reliable indicator of $\mathcal{R} > 1$.

3 Further evidence of dependence of first subband with spin-orbit interaction in magnetic field

In this section we include Zeeman spin-splitting measurements on additional QPCs, source-drain bias, and additional analysis in the small energy limit $\tilde{B}$ and higher subbands.

Additional Zeeman spin-splitting measurements

Additional Zeeman spin-splitting measurements on hole QPCs have been presented here to provide further evidence for the first 1D hole subband behaviour in magnetic field described in the main text.

Supplementary Figure 1 shows the conductance waterfall plots and transconductance colour maps for hole QPCs 4-6 evolving in magnetic field. The data is shown for subbands $n = 1..3$. Subbands $n = 2, 3$ spin split in magnetic field, while subband $n = 1$ has a 0.7 anomaly that evolves to $0.5 \times 2e^2/h$ in magnetic field in field $B > 5\,\mathrm{T}$ [indicated by the green arrows in **panels (a,c,e)**]. In transconductance, subbands $n \geq 2$ Zeeman spin-split, while the first subband remains unaffected by the magnetic field [**panels(b, d, f)**]. The 0.7 anomaly is indicated by the black arrows on the right side of **panels(b, d, f)**.

Supplementary Figure 2 shows the angular response of the first subband in $8\,\mathrm{T}$ of magnetic field in hole QPC 2. Similarly to **Fig. 4f**, the data has been trimmed and reflected around $\varphi/\pi = 0$ for easy comparison with theory. The 0.7 anomaly is broadest around $\varphi/\pi = 0$, and narrows at

$\varphi/\pi = \pm 0.5$ (indicated by the white arrows). The spin gap structures in the top corners of the figure (indicated by the red arrows) are greatly suppressed in QPC 1 due to the small 1D subband spacing and spin-split subband $n = 2$.

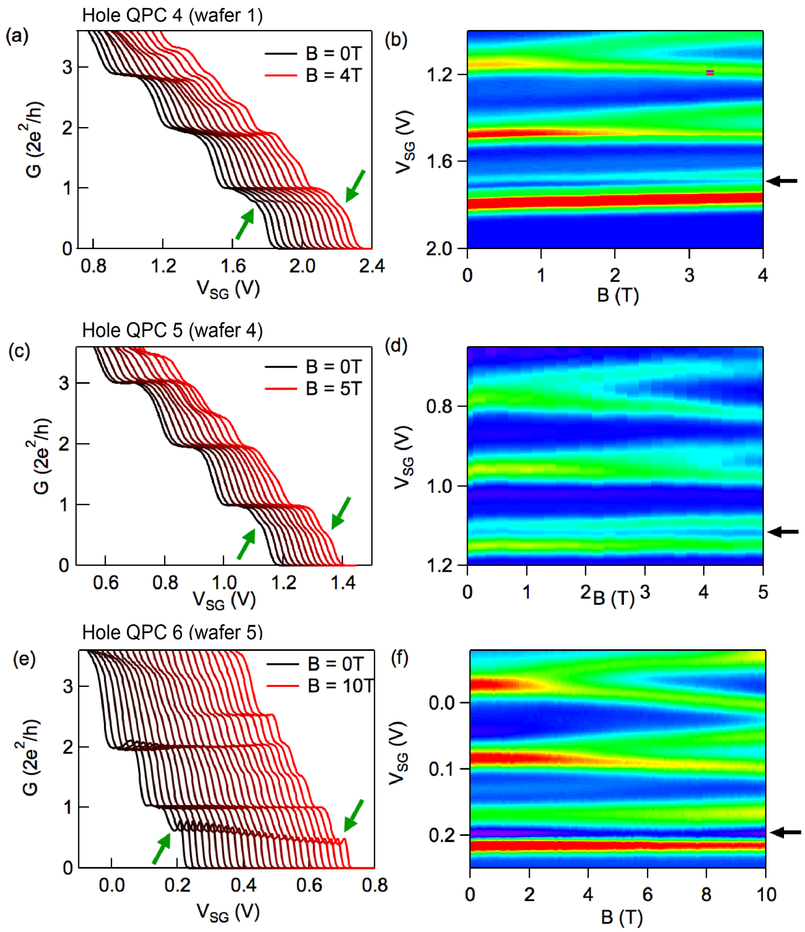

Supplementary Figure 1: (a, c, e) Waterfall plots of the conductance of 1D holes in hole QPCs 4-6 respectively, showing the evolution of the quantization from $2e^2/h$ at $B = 0$T (black trace) to e^2/h in in-plane $B \perp B_{SOI}$ magnetic field up to 10T (red trace). Traces are offset in V_{SG} for clarity. The 0.7 anomaly is indicated with the black arrow for the $B = 0$T trace, and evolves in $0.5 \times G$. (b, d, f) Experimental transconductance colour maps of the Zeeman spin splitting of the first three 1D hole subbands for three different QPC devices. In all cases subbands 2 and 3 spin-split linearly in magnetic field, whereas the first hole subband is only weakly affected by the magnetic field.

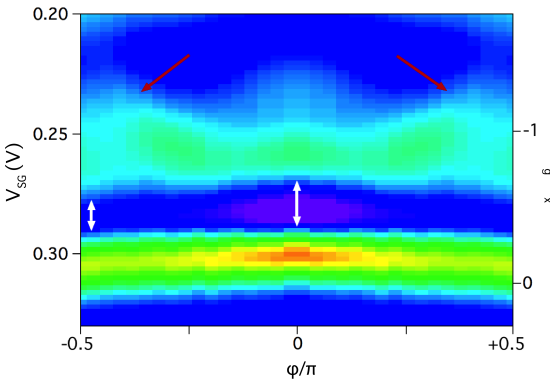

Supplementary Figure 2: Measured transconductance of the first 1D hole subband in hole QPC 2 as a function of magnetic field angle φ where the magnitude of the magnetic field is fixed at $B = 8$ T. Vertical axis is gate voltage scaled by Ω_x, and horizontal axis is magnetic field angle with respect to B_{SOI}. The white arrows indicate the breadth of the 0.7 anomaly. The red arrows indicate the transconductance structures corresponding to the spin gap emerging in the top corners of the figure.

The Zero-Bias Anomaly

The zero-bias anomaly (ZBA) is intimately linked to the 0.7 anomaly [9,10]. To confirm that the feature observed in the hole QPCs has the same origin as the 0.7 anomaly in electron QPCs we show the non-linear differential conductance as a function of source-drain bias for hole QPCs in **Supplementary Figure 3**. At low conductances $G < 0.7 \times 2e^2/h$, there is a peak centred around zero bias, consistent with the 0.7 anomaly and zero bias peak reported in electron QPCs[10].

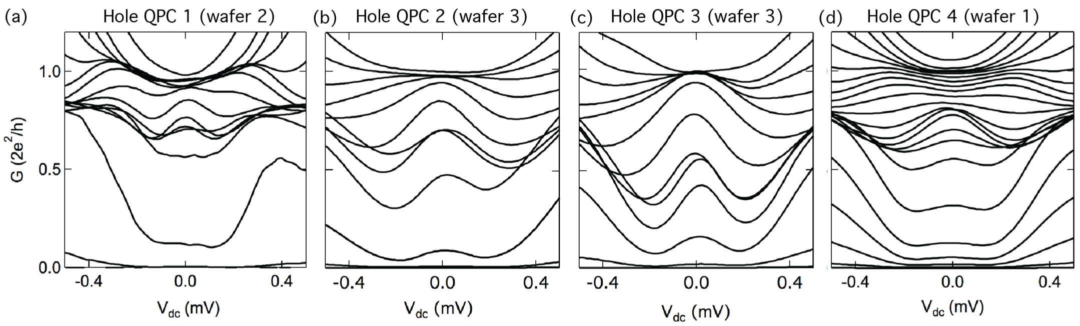

Supplementary Figure 3: Non-linear differential conductance G as a function of dc voltage V_dc for a range of side-gate bias values (or QPC width) for hole QPCs 1-4. In each QPC device there is a conductance peak centered around $V_{dc} = 0$ that splits into two peaks where $0.7 \times (2e^2/h) > G > (2e^2/h)$.

Limit of small energy $\tilde{B}$

Modelling of the first 1D electron subband in Ref [9] using (SOPT) yields the following prediction: that for fixed values of side gate voltage V_{sg}, or 1D confinement, the leading dependence of the non-linear conductance on $\tilde{B}$ is quadratic. The quadratic relation takes the form

$$\frac{g_{nl}(\tilde{B},\tilde{T},\tilde{V}_{sd})}{g_{nl}(0,0,0)} \approx 1 - \frac{\tilde{B}^2}{\tilde{B}_*^2} - \frac{\tilde{T}^2}{\tilde{T}_*^2} - \frac{\tilde{V}_{sd}^2}{\tilde{V}_{sd*}^2} \tag{22}$$

where $\tilde{B}/\tilde{B}_* \ll 1$ and $\tilde{B}_*, \tilde{T}_*$ and $\tilde{V}_{sd*}$ are V_{sg} dependent crossover scales that govern the strength of the 0.7 anomaly for finite exchange interaction energies. We demonstrate here that the relationship holds for the hole 0.7 anomaly with SOI in **Supplementary Figure 4**.

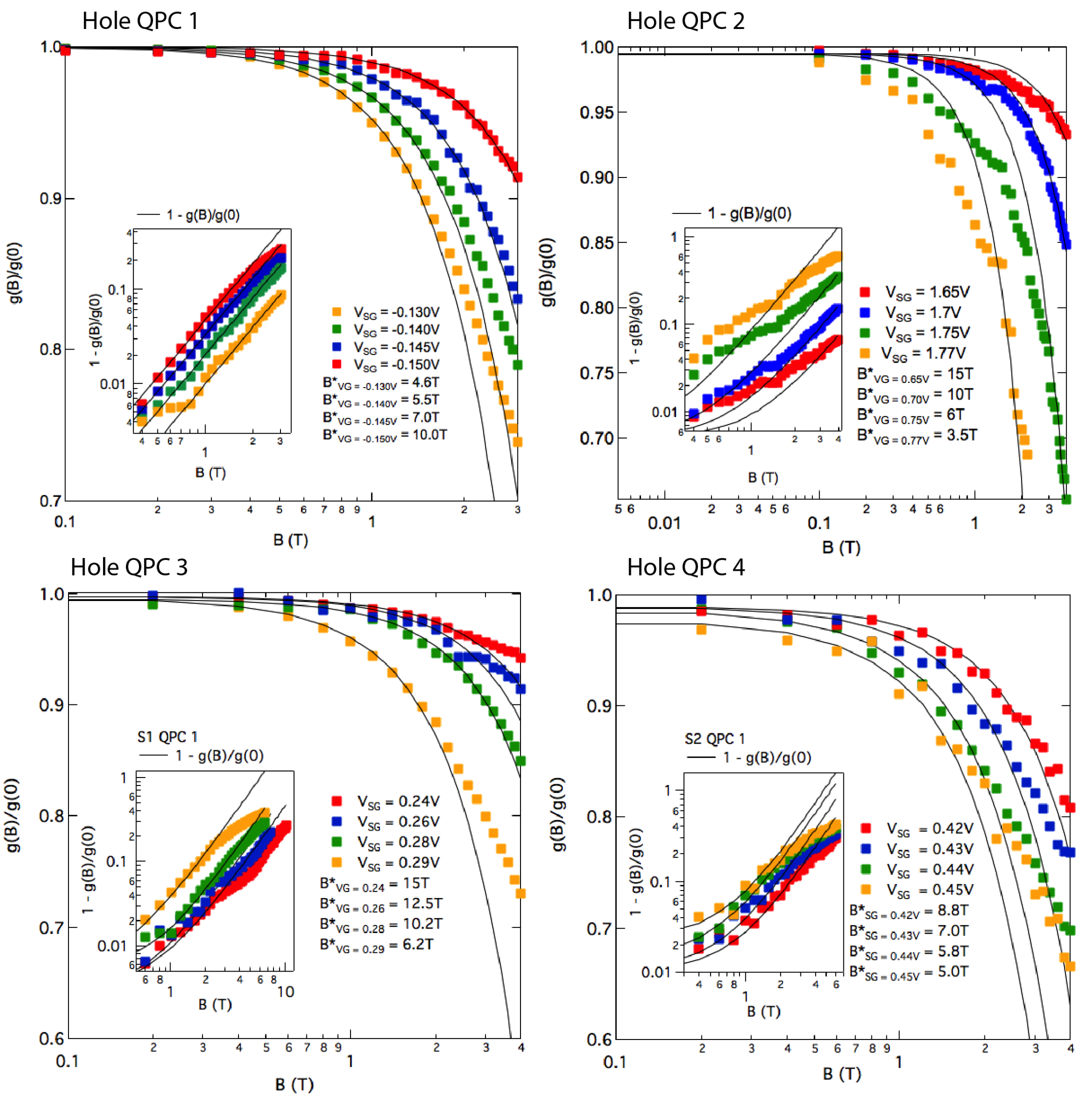

Supplementary Figure 4: $g(B)/g(0)$ as a function of B for a range of top gate voltages (densities) for hole QPCs 1-4 in (a-d) respectively. Vertical axis is normalised conductance $g(B)/g(0)$ plotted against logarithmic B scale. Insets: logarithmic conductance $1 - g(B)/g(0)$ plotted against logarithmic B, following the quadratic relation in equation (22).

Proximity of the second subband to the first subband

In the main text we discuss the absence of a spin gap minima on the first 1D hole conductance plateau for the traces shown in **Figures 1f-g** and **Supplementary Figure 1a,c,e** with reference to the transconductance colour maps in **Figure 4**. Here we directly compare the calculated conductance with the measured conductance for both electrons and holes as a function of scaled gate voltage V_g/Ω_x in **Supplementary Figure 5**.

The calculated conductance is shown for electrons in **Supplementary Figure 5a**, where $U = 0.1$ and $R = 0$ for magnetic field $B = 0\,\mathrm{T}$ and $B = 4\,\mathrm{T}(\approx 0.4\Omega_x)$. The measured conductance for 1D electrons is shown in **Supplementary Figure 5b** for magnetic field $B = 0\,\mathrm{T}$ and $B = 4\,\mathrm{T}(\approx 0.4\Omega_x)$. The model in **Supplementary Figure 5a** is purely 1D and describes only one subband, while **Supplementary Figure 5b** shows two conductance plateaus over the same energy scale.

Particular care must be taken when comparing the purely 1D model to measured conductance in the case of 1D holes with SOI and 0.7 anomaly. In **Supplementary Figure 5c**, the calculated conductance at $R = 1.26$ shows the 0.7 anomaly structure (indicated by the green arrow), and the spin gap structure (indicated by the blue arrow) on the first plateau. The first 'riser' containing the 0.7 structure extends over a gate range of $\Delta V_g/\Omega_x \sim 1$, while the spin gap structure extends over a much further range of $\Delta V_g/\Omega_x \sim 3$. We compare the calculated conductance with our (scaled) measured conductance in **Supplementary Figure 5d** and find that the second $n = 2$ subband riser emerges right where we expect the spin gap conductance minima to occur based on **Supplementary Figure 5c**.

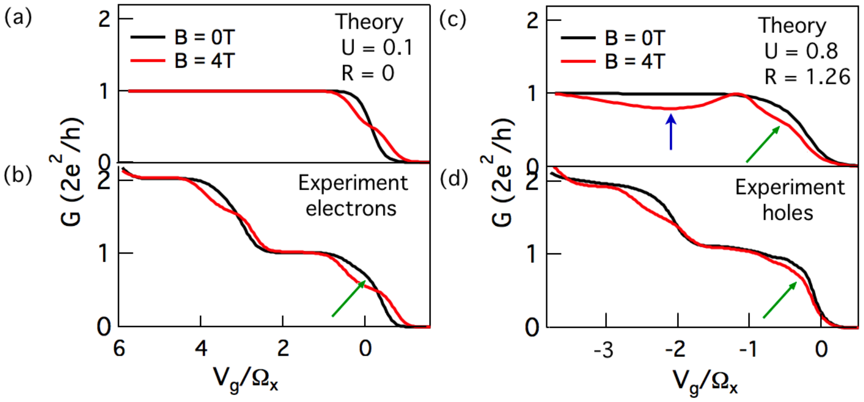

Supplementary Figure 5: (a) Calculated conductance of the first 1D subband for weakly interacting electrons with no SOI. When the magnetic field is increased a half step forms in the conductance trace (red) that crosses the zero-field conductance trace (black). (b) The experimental conductance trace where the spin-resolved conductance trace crosses the zero-field trace similarly to the theoretical model in (a). (c) Calculated conductance of the first 1D subband for strongly interacting holes with strong SOI. The spin-resolved conductance trace (red) does not cross the zero-field trace (black). The 0.7 anomaly is evident at low energy and an emerging dip in the conductance at higher energy is indicative of the opening of the spin gap. (d) The experimental conductance trace where the spin-resolved conductance trace does not cross the zero-field conductance trace, consistent with the theoretical model in (c). The 0.7 anomaly is also evident (indicated by the green arrow). A second shoulder in the conductance above the 0.7 anomaly (indicated by the black arrow) suggests the weak emergence of a dip in conductance due to the opening of the spin gap.

Anisotropy of the in-plane hole g-factor

In the main text we make note that the anisotropy of the first 1D hole subband ‘splitting’ (that is, the ‘size’ of the 0.7 anomaly in gate voltage (energy)), has the opposite anisotropy to the Zeeman spin-splitting of the higher 1D hole subbands. In **Supplementary Figure 6** we present for the reader’s verification the complete transconductance colour map of $n = 1..7$ subbands as a function of magnetic field angle with respect to the spin-orbit field B_{SOI} (or QPC channel). The first subband has a broad (in V_{SG}) 0.7 anomaly at $\varphi/\pi = 0, 1.0$ (or $B \parallel B_{SOI}$); in contrast subbands $n = 2..7$ have minimal spin splitting. When $\varphi/\pi = 0.5$ (or $B \perp B_{SOI}$), the opening of the spin gap coincides with the 0.7 anomaly becoming narrower, while subbands $n = 2..7$ are Zeeman spin-split.

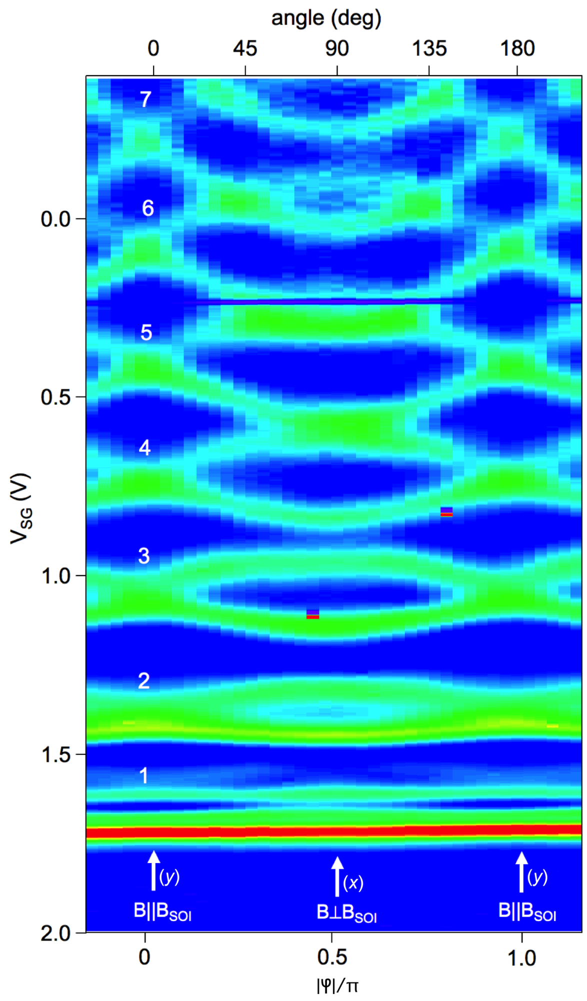

Supplementary Figure 6: Complete transconductance map (of data presented in **Figure 4** in the main text) plotted against V_{SG} and magnetic field angle φ (angle in degrees provided on top axis), where $|B| = 4\,\mathrm{T}$. Magnetic field orientation with respect to the spin-orbit field B_{SOI}, and the corresponding x, y-directions, are indicated at the bottom of the colour map. Subbands are labelled $1..7$ in white just above the subband at the point of minimal spin-splitting on the left side of the colour map.

Zeeman spin-splitting of the first 1D hole subband in the out-of-plane z-direction

The theory in Ref [1] assigns the direction of the spin orbit field B_{SOI} to in-plane perpendicular with the QPC (y-direction). The field will couple to B_{SOI} when the applied magnetic field has a component either parallel to the QPC (x-direction) and/or aligned out of the plane of the 2DHG (z-direction). In the main text of the paper we examined the first subband in the plane of the of the 2DHG (x, y-directions). Here we briefly comment on the first subband in an out-of-plane (z-direction) field.

The quantisation axis for holes confined to a 2D plane is along the z-direction; light-hole-heavy-hole (LH-HH) mixing leads to a small in-plane component. The out-of-plane tensor component g_{zz} has been measured to be in the range of $5 - 7.2$ [11,12], consistent with theory [13–15]. (The in-plane g-factors are an order of magnitude smaller, with $g_{xx} \approx 0.5$ and g_{yy} generally too small to resolve in measurements [16].

In **Supplementary Figure 7**, the measured transconductance is shown for $B \perp 2DHG$, the out-of-plane field direction. At relatively low magnetic field $B < 1\,\mathrm{T}$, the large out-of-plane g_{zz}-factor gives rise to large Zeeman spin-splitting. This field orientation also couples to the orbital momentum of the subbands, resulting in an upward curvature of the subbands. As B increases, the magnetic length of the holes shrinks and eventually becomes smaller than the width of the 1D constriction, and the higher subbands one by one enter the quantum Hall regime.

When the magnetic field is oriented along the out-of-plane z-direction, it is also $B \perp B_{SOI}$

and should open a spin-gap in the first 1D hole subband. This opening of the spin-gap is consistent with the absence of spin-splitting in the first 1D hole subband up to $0.9\,\mathrm{T}$ **Supplementary Figure 7**. This behaviour has also been observed in Ref [17] where there is an absence of spin-splitting up to $\sim 3\,\mathrm{T}$, and in Ref [11] up to $\sim 2\,\mathrm{T}$. At higher field, the first 1D hole subband abruptly begins to spin-split, and the size of the spin-splitting becomes large quickly.

At low magnetic field, the spin-orbit energy E_{SOI} is larger than the Zeeman energy E_Z, and the first subband is insensitive to magnetic field. The sudden transition to large spin-splitting occurs when $E_Z > E_{SOI}$. In this regime, the spin gap in the dispersion relation has become so large as to resemble the usual parabolic dispersion relation once more, and the bands become sensitive to magnetic field once more (see **Supplementary Figure 8**).

Once the Zeeman energy overwhelms the spin-orbit gap, $g\mu_B B \gg E_{SOI}$, we expect the first 1D hole subband to Zeeman spin-split. In our 1D hole QPCs, $E_{SOI} \approx 500\,\mu\mathrm{eV}$ and $g_{zz} \sim 5$ [12]. We therefore do not expect to observe Zeeman spin-splitting of the first 1D hole subband until $B > 1.7\,\mathrm{T}$. This is a higher magnetic field than was accessible in our experiments for this work.

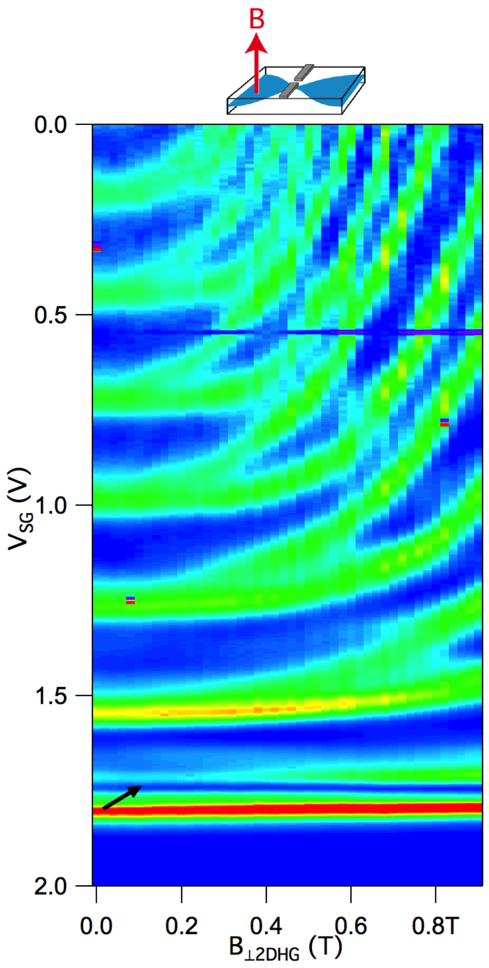

Supplementary Figure 7: Transconductance colour map of Zeeman spin-splitting of the 1D subbands in hole QPC 4 in magnetic field aligned perpendicular to the QPC and out of the 2DHG plane (see schematic above panel). The subbands $n = 2..6$ strongly spin split in magnetic field, while the first subband in both panels remains unchanged. The position of the 0.7 anomaly is indicated by the black arrows.

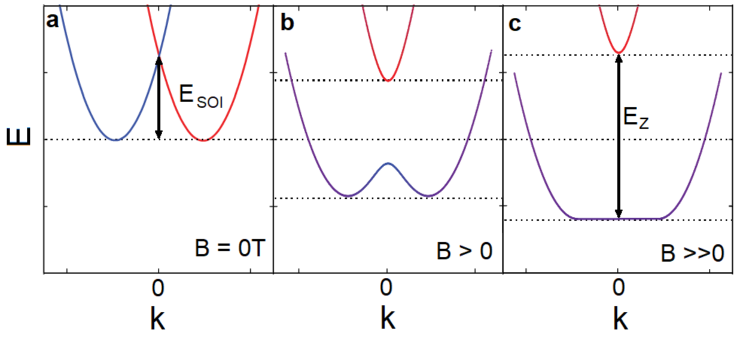

Supplementary Figure 8: (a) 1D parabolic dispersion relation spin-split in k due to SOI. (b) Applied magnetic field results in spin gap opening in 1D dispersion relation at $k = 0$. $E_{SOI} > E_Z$. (c) Large applied magnetic field results in $E_Z > E_{SOI}$ and the bands resemble the generic 1D dispersion relation again.

4 Fabrication and measurement methods

The following includes information on the device fabrication and electrical set-up for the measurements presented in the Main Text and Supplementary Information.

Electron QPC device AuGe was used for the ohmic contacts to the 2DEG. A $20\,\mathrm{nm}$ thick layer of Al_2O_3 was used to insulate the Ti/Au top gate electrode from the ohmic contacts. Electrical measurements were performed at a temperature of $300\,\mathrm{mK}$ using standard AC lock-in techniques with an excitation voltage of $50\,\mu\mathrm{V}$ at a frequency of $7\,\mathrm{Hz}$. QPC electron densities, surface gate dimensions and 2DEG depth are summarised in Supplementary Table 1.

Hole QPC devices AuBe was used for the ohmic contacts to the 2DHS. A $20\,\mathrm{nm}$ thick layer of Al_2O_3 was used to insulate the Ti/Au top gate electrode from the ohmic contacts. Electrical measurements were performed at a temperature of $40\,\mathrm{mK}$ using standard AC lock-in techniques with an excitation voltage of $100\,\mu\mathrm{V}$ at a frequency of $7\,\mathrm{Hz}$. QPC hole densities, surface gate dimensions and 2DHG depth are summarised in Supplementary Table 1. QPC 1 has hole mobility of $\mu = 0.1 \times 10^6\mathrm{cm}^2\,\mathrm{V}^{-1}\,\mathrm{s}^{-1}$. QPCs 2-6 have hole mobilities $\mu = 0.5 \times 10^6\mathrm{cm}^2\,\mathrm{V}^{-1}\,\mathrm{s}^{-1}$

1. Goulko, O., Bauer, F., Heyder, J. & von Delft, J. Effect of spin-orbit interactions on the 0.7 anomaly in quantum point contacts. *Phys. Rev. Lett.* **113**, 266402 (2014).
2. Datta, S. *Electronic Transport in Mesoscopic Systems* (Cambridge University Press, 1995).
3. Wetterich, C. Exact evolution equation for the effective potential. *Physics Letters B* **301**, 90 – 94 (1993).
4. Birkholz, J. E. & Meden, V. Spin-polarized currents through interacting quantum wires with nonmagnetic leads. *Phys. Rev. B* **79**, 085420 (2009).
5. Birkholz, J. E. *Spin-orbit interaction in quantum dots and quantum wires of correlated electrons – A way to spintronics?* Ph.D. thesis, Georg-August-Universität zu Göttingen (2008).
6. Bauer, F. *0.7 Anomaly of Quantum Point Contacts: Treatment of Interactions with Functional Renormalization Group.* Master's thesis, LMU-München (2008).
7. Metzner, W., Salmhofer, M., Honerkamp, C., Meden, V. & Schönhammer, K. Functional renormalization group approach to correlated fermion systems. *Rev. Mod. Phys.* **84**, 299–352 (2012).
8. Bauer, F., Heyder, J. & von Delft, J. Functional renormalization group approach for inhomogeneous interacting fermi systems. *Phys. Rev. B* **89**, 045128 (2014).
9. Bauer, F. *et al.* Microscopic origin of the '0.7-anomaly' in quantum point contacts. *Nature* **501**, 73–78 (2013).

10. Cronenwett, S. M., Oosterkamp, T. H. & Kouwenhoven, L. P. A tunable Kondo effect in quantum dots. *Science* **281**, 540–544 (1998).

11. Nichele, F. *et al.* Characterization of spin-orbit interactions of GaAs heavy holes using a quantum point contact. *Phys. Rev. Lett.* **113**, 046801 (2014).

12. Srinivasan, A. *et al.* Using a tunable quantum wire to measure the large out-of-plane spin splitting of quasi two-dimensional holes in a gaas nanostructure. *Nano Letters* **13**, 148–152 (2013).

13. Winkler, R. *Spin-orbit coupling effects in two-dimensional electron and hole systems*. Springer tracts in modern physics (Springer, Berlin, 2003).

14. Zülicke, U. Electronic and spin properties of hole point contacts. *physica status solidi c* **3**, 4354–4358 (2006).

15. Simion, G. E. & Lyanda-Geller, Y. B. Magnetic field spectral crossings of Luttinger holes in quantum wells. *Phys. Rev. B* **90** (2014).

16. Chen, J. C. H. *et al.* Observation of orientation- and k -dependent Zeeman spin-splitting in hole quantum wires on (100)-oriented AlGaAs/GaAs heterostructures. *New Journal of Physics* **12**, 033043 (2010).

17. Komijani, Y. *et al.* Anisotropic Zeeman shift in p-type GaAs quantum point contacts. *EPL (Europhysics Letters)* **102**, 37002 (2013).
